# A microRNA molecular modeling extension for prediction of colorectal cancer treatment

**DOI:** 10.1186/s12885-015-1437-0

**Published:** 2015-06-18

**Authors:** Jian Li, Ulrich R. Mansmann

**Affiliations:** 1 Institute for Medical Informatics, Biometry and Epidemiology, Ludwig-Maximilians-University München, Munich, Germany; 2 German Cancer Consortium (DKTK), Heidelberg, Germany; 3 German Cancer Research Center (DKFZ), Heidelberg, Germany

**Keywords:** MicroRNA regulation, Signaling pathway, Individualized medicine, Colorectal cancer

## Abstract

**Background:**

Several studies show that the regulatory impact of microRNAs (miRNAs) is an essential contribution to the pathogenesis of colorectal cancer (CRC). The expression levels of diverse miRNAs are associated with specific clinical diagnoses and prognoses of CRC. However, this association reveals very little actionable information with regard to how or whether to treat a CRC patient. To address this problem, we use miRNA expression data along with other molecular information to predict individual response of CRC cell lines and CRC patients.

**Methods:**

A strategy has been developed to join four types of information: molecular, kinetic, genetic and treatment data for prediction of individual treatment response of CRC.

**Results:**

Information on miRNA regulation, including miRNA target regulation and transcriptional regulation of miRNA, in integrated into an *in silico* molecular model for colon cancer. This molecular model is applied to study responses of seven CRC cell lines from NCI-60 to ten agents targeting signaling pathways. Predictive results of models without and with implemented miRNA information are compared and advantages are shown for the extended model. Finally, the extended model was applied to the data of 22 CRC patients to predict response to treatments of sirolimus and LY294002. The *in silico* results can also replicate the oncogenic and tumor suppression roles of miRNA on the therapeutic response as reported in the literature.

**Conclusions:**

In summary, the results reveal that detailed molecular events can be combined with individual genetic data, including gene/miRNA expression data, to enhance *in silico* prediction of therapeutic response of individual CRC tumors. The study demonstrates that miRNA information can be applied as actionable information regarding individual therapeutic response.

**Electronic supplementary material:**

The online version of this article (doi:10.1186/s12885-015-1437-0) contains supplementary material, which is available to authorized users.

## Background

With an average of 610,000 deaths per year, CRC has become the second most common cause of cancer death on a global scale. Because it is most commonly diagnosed at advanced stages, approximately 50 % of patients diagnosed with CRC will surrender to the disease [1]. From a molecular perspective, CRC is characterized by the accumulation of genetic alterations affecting the cellular functionalities of oncogenes and tumor suppressor genes, leading to genomic instability and cellular dysfunction [2]. A number of deregulated signaling pathways, most notably Wnt [3], Notch [4], Hedgehog [5] and others [6, 7], have been identified as maintaining the malignant cellular growth and cancerous functional integration of CRC. The signaling network based on these deregulated pathways steers the oncogenetic development. Furthermore, miRNAs are deeply involved in the pathogenesis of CRC by affecting key components of those signaling pathways. They play significant roles in regulating cell growth, proliferation, invasion and metastasis in CRC [8–14]. Moreover, recent studies have demonstrated that miRNAs detected in blood serum, plasma and even in stool offer novel non-invasive approaches to diagnose CRC [13, 15–17]. Other recent studies revealed experimentally that expression levels of certain miRNAs were associated with specific prognosis and therapeutic outcomes in CRC, which provides compelling evidence that miRNAs have the potential to be prognostic and predictive biomarkers [18–21]. Further, detailed molecular information related to miRNA function in clinical application was reported in the study of Melo & Kalluri [22]. Our study introduces an *in silico* model for the colorectal cancer cell which implements miRNA information and explores its potential for improved response prediction to specific treatment.

## Methods

### Colorectal Cancer Patients

The gene-expression and miRNA-expression data of the 22 CRC patients examined in this study can be downloaded from https://tcga-data.nci.nih.gov/tcga/, which is provided by the Cancer Genome Atlas, with the following filter setting configuration: Select a disease → *COAD- Colon adenocarchinoma*; Data type → *RNASeqV2/miRNASeq*; Center/Platform *→ All*; Batch number → *All*; Sample → *Patient ID*; Data level → *Level 3*; Availability → *None*.

Each dataset was produced through the analysis of high-quality colon tumor samples from the participants. Each dataset was normalized using the trimmed mean of M-values normalization method proposed by Robinson & Oshlack [23], to remove systematic technical effects and minimize the sequencing technical bias on the data.

### The Genomic Data of CRC Cancer Cell Lines from NCI-60

The CRC cancer cell lines examined in this study are COLO-205, HCC-2998, HCT-116, HCT-15, HT29, KM12, SW-620. The gene-expression data of these cancer cell lines can be downloaded via [24]. The miRNA-expression data can be accessed via [25].

### The Non-Steroidal Anti-Inflammatory Drug (NSAID) Model

Our previous study [26] introduced the *in silico* NSAID model, which incorporates information of 20 diverse CRC-relevant signaling pathways, such as Wnt, Notch, BMP, beta-catenin and Hedgehog, and other molecular features. The model contains different types of biological components such as genes, RNAs, proteins and complexes. Components of the network are used to quantify specific aspects of tumorigeneity in terms of the cancer hallmarks [27]. The study demonstrated application for two therapeutic developmental strategies: synthetic lethality and miRNA biomarker discovery.

### Molecular Addition of miRNA-Regulation (miRAO)

**Fig. 1:**
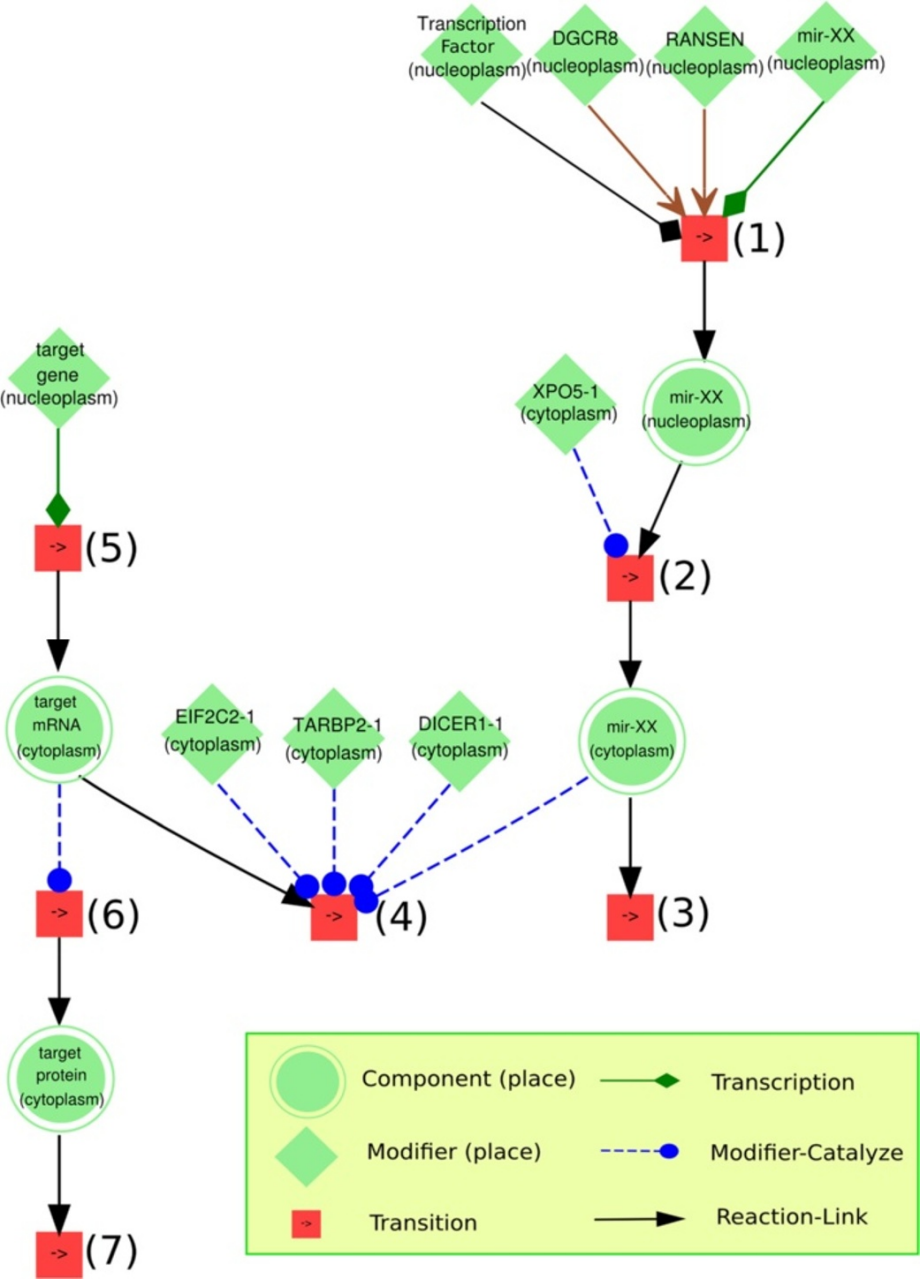
The conceptual visualization of miR-add-on algorithm. The step (1) simplifies two biological processes: **(a)** the transcription catalyzed by transcriptional activator or repressor (if available); **(b)** the primary transcript (pri-miRNA) is cropped into a hairpin intermediate (pre-miRNA) by the nuclear 650 kDa microprocess complex, which consists of humans of the RNase III DROSHA (RNASEN) and the DiGeorge syndrome critical region gene 8 (DGCR8) . The step (2) defines a transport reaction to translocate the miRNA into cytoplasm so that it is ready for the following target binding process. The step (3) is a degradation process. The step (4) simplifies two processes: **(a)** mature miRNA binds to different protein partners and turns into the RNA-induced silencing complex (RISC); **(b)** RISC recognizes the target mRNA and binds to it. The steps (5), (6) and (7) are transcription, translation and degradation of target gene, mRNA and protein, respectively

The algorithmic basis for our study is the NSAID model introduced by Li & Mansmann [26]. The following steps implement miRNA regulations:

1. Convert miRNA-target data into a data array (miRT) (with Ensembl-ID as key; miRNA-ID and references (PubMed) as value); for example, miRT[ENSG00000236342] = (mir-1238, 17964270).
2. Convert the TransmiR data into a data array (TFmiR) (with miR-ID as key; Ensembl-ID, transcriptional regulation, references (PubMed) as value); for example, TFmiR[mir-223] = (ENSG00000159216, repression, 17996649).
3. Divide the NSAID model (XML file of NSAID) into different data arrays according to component type (such as gene array, mRNA array, protein array, reaction array, etc.)
4. Iterate all the gene components in the gene array; when a gene with Ensembl-ID matches a key of miRT, then the corresponding miRNA is created in the model by defining name, ID, location and other. Afterwards, the TFmiR is applied to identify the transcriptional factors that regulate the expression level of this miRNA. The corresponding type of transcription reaction is defined to link the miRNA gene with the miRNA. Afterwards, the miRNA is translocated into cytoplasm and modeling of its regulation on the corresponding mRNA is created and added into the model (Fig. 1). (The detailed molecular modeling is explained in the study of Li et al. [28].)

### Model Initialization with miRNA and Gene Expression Data

The initial value of all components in the model is zero. The miRNA- and gene-expression data is available through the link from the Cancer Genome Atlas (as mentioned in the paragraph “Colorectal Cancer Patients”) or from the cell line data. These datasets are converted into a data array (similarly to the method explained above). The keys of this data array are the miRNA-IDs and Ensembl-IDs as well as the expression values. Afterwards, the expression levels of miRNA genes and normal genes in the model are set to the values as given by the miRNA-ID and Ensembl-ID. During Petri net simulation, signal fluxes of reactions are simulated in the model by transcription reactions and are expanded to the rest of the model by other reactions defined in the model. This data initialization is the input for the Flux Comparative Analysis explained in the following.

### Mathematical Implementation of Sirolimus and LY294002

We study the effect of sirolimus as a specific mTor inhibitor. According to Nashan [29], its dissociation constant is 0.65 nM indicating an inhibition of biochemical reactions catalyzed by the mTor protein complex. LY294002 is a potent inhibitor of PI3K, which catalyzes the conversion from PIP2 to PIP3. The dissociation constant of LY294002 is 210 nM [30]. Table 1 contains the molecular modeling of both drugs and the corresponding control state (without treatment).

**Table 1:** 'E': enzyme; 'I': Inhibitor. Currently, we have only applied the mass action law for implementing biochemical reaction

Sirolimus treatment	CRC patient + sirolimus	CRC patient
Molecular modeling	mTor complex II (E); Sirolimus (I)	mTor complex II (E)
	PRKCG + ATP → → → P-PRKCG + ADP	PRKCG + ATP → → → P-PRKCG + ADP
	mTor complex II (E); Sirolimus (I)	mTor complex II (E)
	SGK + ATP → → → P-SGK + ADP	SGK + ATP → → → P-SGK + ADP
	mTor complex II (E); Sirolimus (I)	mTor complex II (E)
	PRKCA + ATP → → → P-PRKCA + ADP	PRKCA + ATP → → → P-PRKCA + ADP
	mTor complex II (E); Sirolimus (I)	mTor complex II (E)
	AKT + ATP → → → P-AKT + ADP	AKT + ATP → → → P-AKT + ADP
	mTor complex II (E); Sirolimus (I)	mTor complex II (E)
	HIF1A + ATP → → → P-HIF1A + ADP	HIF1A + ATP → → → P-HIF1A + ADP
	mTor complex II (E); Sirolimus (I)	mTor complex II (E)
	PPARGC1 + ATP → →P-PPARGC1 + ADP	PPARGC1 + ATP → P-PPARGC1 + ADP
	mTor complex II (E); Sirolimus (I)	mTor complex II (E)
	EIF4EBP + ATP → → P-EIF4EBP + ADP	EIF4EBP + ATP → →P-EIF4EBP + ADP
	mTor complex II (E); Sirolimus (I)	mTor complex II (E)
	PPARG + ATP → → P-PPARG + ADP	PPARG + ATP → → P-PPARG + ADP
LY294002 treatment	CRC patient + LY294002	CRC patient
Molecular modeling	Enzymes; LY294002 (I)	Enzymes
	PIP2 + ATP → → → PIP3 + ADP	PIP2 + ATP → → → PIP3 + ADP

For instance, the substance A and B participate in a reaction catalyzed by an enzyme and inhibitor to produce the products C and D: enzyme; inhibitor

A + B → → → C + D

where the mathematical implementation: [C] = [D] = [A] * [B] * [enzyme] * [iKd] / [inhibitor] * [eKd], eKd: enzymatic dissociation constant; iKd: inhibitor dissociation constant

### Flux Comparative Analysis (FCA)

The FCA is an advanced Petri net simulation strategy. As the name suggests, it is an analysis of flux comparison of two different states of a molecular model. The goal of FCA analysis is to detect whether a therapeutic intervention (drug treatment) can cause a significant flux change with regard to the structure of an entire molecular network, in order to predict how an individual would respond to a therapeutic intervention [26]. Essentially, during FCA, two states are generated for each cell line/patient for each treatment: one is the control state (without treatment) and the other is the perturbation state (with treatment). During the Petri net simulation, the fluxes generated for each state in the model are compared for each patient. The following simulation algorithm code is applied to generate the steady state of each state:

- R_i_ = the i-th reaction in the molecular model; Parameters of R_i_ include speed (S), kinetic parameter (k), product (p), reactant (a), enzyme (e)
- C_j,t_ = the concentration of the j-th bio-object (such as gene, protein) in the model at time step t
- S_t_ = C_a,t_ * C_e,t_ * k
- N, M = the number of reactions and bio-objects in the model, respectively.

1. Input: Gene-expression data and miRNA-expression data
2. For each j (from 1 to M) at time step t:
3. if C_j,t_ – C_j,t-5_ > 0.001:
4. then reachSteadyState = False
5. If not reachSteadyState:
6. for each i (from 1 to N) at time step t:
7. if C_p,t-1_ < C_a,t-1_ & S_t_ < C_a,t-1_ * 0.75:
8. then evaluate R_i_ as Petri net firing rule at t:
9. C_p,t_ = C_p,t-1_ + S_t_
10. C_a,t_ = C_a,t-1_ - S_t_
11. If reachSteadyState:
12. select the readout components
13. Output: compare readout components between two states (Control vs. Treatment)

### Definition of Sensitivity Score for Drug Response

*Experimentally based sensitivity score* (experimental data) = GI_50_

(GI_50_: the -log mol/L drug/concentration yielding a growth inhibition of 50 %, [31])

*Model-based sensitivity score* (prediction data) = log(P) + K

(P: relative change value of readout component 'proliferation' hallmark in the treatment state compared to that in the control state. In this case, the hallmark 'proliferation' is selected as the readout component for the FCA analysis; K: constant value, currently estimated as 5.2. the hallmark "proliferation", as a mirror of proliferative ability, is taken as the primary outcome, since the cell line models quantify response on treatment by its impact on cellular growth)

### Correlation Between Experimentally Measured and Computationally Simulated Scores

It is of interest to calculate the Pearson correlation between observed and predicted response of cell lines under a specific treatment. Aggregating correlation over all treatments was calculated following the principles of Bland and Altman as presented in [32].

### The Availability of the Model

The XML file of the NSAID-miR model is available under [33].

## Results

**Fig. 2:**
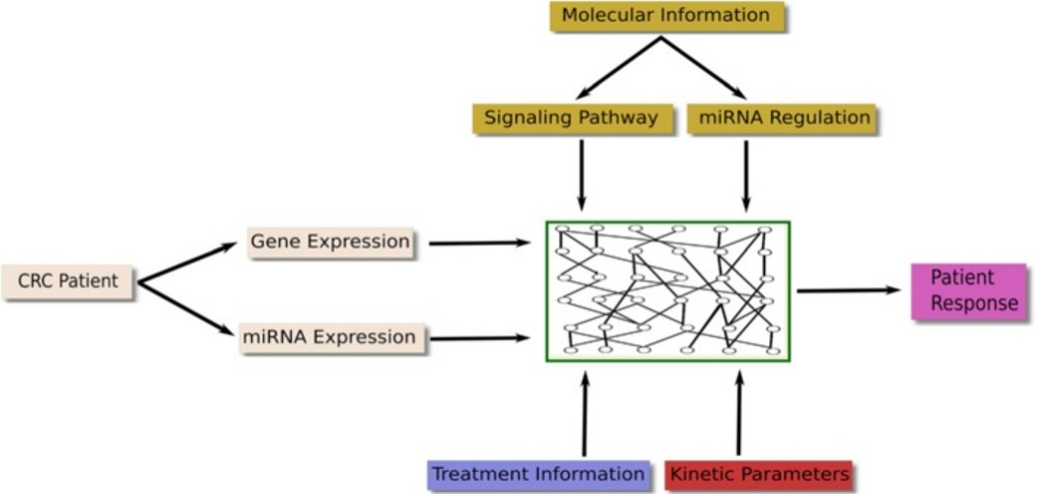
The work-flow of the molecular concept for individualized medicine. In order to reflect or capture the individual patient response to specific treatment, four types of information are currently needed as input to construct a molecularly based model, which might act as a 'Virtual Patient' to achieve the goal of individualized medicine

A strategy for prediction of individual treatment response is proposed which is based on an *in silico* environment in which the molecular regulation effect of miRNAs combined with other molecular information can be utilized. A flowchart depicts the work-flow of this concept (Fig. 2). There are four major sources of input information: molecular, kinetic, individual genetic (miRNA/mRNA expression data) and treatment data.

### The microRNA Extension for the Non-Steroidal Anti-Inflammatory Drug (NSAID) Model

The NSAID model depicts a consolidated molecular basis of CRC which is extended in this study by including miRNA regulation. An algorithm, the miRNA-add-on (miRAO), is proposed which automatically adds miRNA regulation into molecular models such as the NSAID model. The miRAO checks whether each miRNA has been validated with targets according to a specified miRNA-target database (Additional file 1) and whether each miRNA has validated transcription factors (TFs) according to the TransmiR database (version 1.2), which provides detailed information regarding type and effect of transcriptional regulations on miRNAs with corresponding literature [34]. If such validated gene targets or miRNA transcription factors are available, then the miRAO adds the molecular miRNA regulation to the model (Fig. Methods;). In this way, the NSAID model is extended with available validated miRNA-target and TF-miRNA information, which strengthens the model with detailed molecular regulation mechanisms related to miRNA. The new version of the model is named NSAID-miR; a summarization is given in Table 2.

**Table 2:** The component/reaction summary of the NSAID-miR model

Component	No.	Reaction	No.
Gene	1284	Transcription	1933
mRNA	2360	Translation	898
Protein	1473	Decay	2172
miRNA	367	Complex-formation	579
Compound	44	Translocation	1361
Complex	856	Phosphorylation	749
Pseudo-object	21	Dephosphorylation	357
SiRNA	1	Activation	341
		miRNA-binding	1516
Sum:	6406	Sum:	9906

### Therapeutic Prediction of Ten Signaling Agents on CRC Cancer Cell Lines

**Fig. 3:**
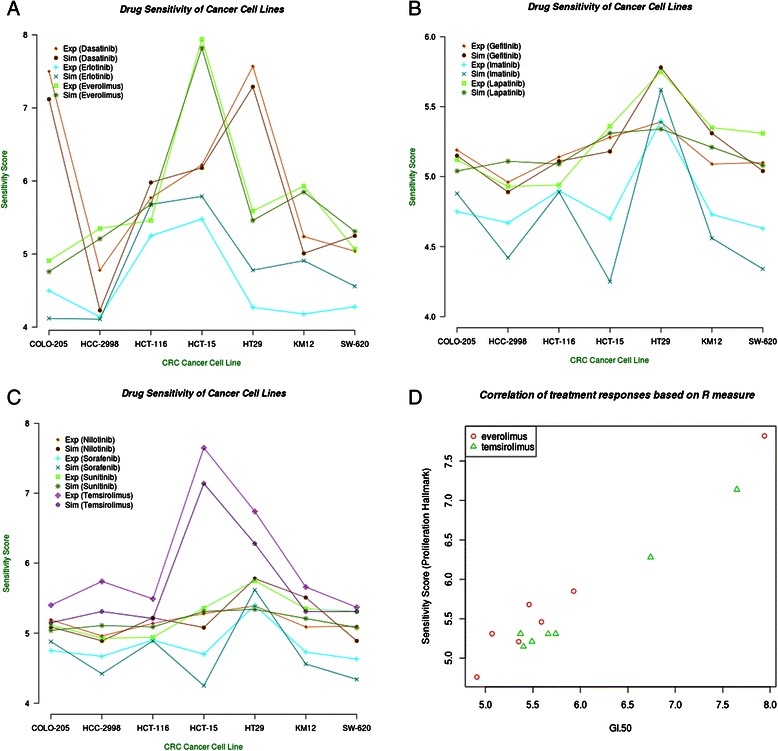
Sensitivity scores of CRC cancer cell lines. **a**: the plot visualizes how CRC cancer cell lines respond to the treatment of dasatinib, erlotinib, and everolimus. **b**: the plot quantitatively displays how CRC cancer cell lines respond to the treatment of gefitinib, imatinib and lapatinib. **c**: the plot quantitatively shows how CRC cancer cell lines respond to the treatment of nilotinib, sorafenib, sunitinib and temsirolimus. All data are attached in the Additional file 2 (Exp: experimentally based sensitivity score; Sim: model-based sensitivity score). **d**: R^2^ measure between responses (GI-50) of CRC cell lines and predicted sensitivity scores to treatments of everolimus and temsirolimus

In order to validate the NSAID-miR model, the inhibition effects of ten signaling agents on seven CRC cancer cell lines are simulated and compared to experimentally measured inhibition effects (sensitivity scores) from the study of Holbeck et al. [31]: COLO-205, HCC-2998, HCT-116, HCT-15, HT29, KM-12, and SW-620. The NSAID-miR model is initialized with gene-expression and miRNA-expression data of individual CRC cancer cell lines (Methods). The effects of ten signaling agents are studied: dasatinib, erlotinib, everolimus, gefitinib, imatinib, lapatinib, nilotinib, sorafenib, sunitinib, and temsirolimus. The inhibition potential of these tyrosine-kinase inhibitors can be specified through corresponding dissociation constants. They were measured experimentally by Karaman and colleagues [35]. How these inhibition effects are modeled is described in Table 3. Subsequently, we performed the FCA to calculate the simulated sensitivity scores of the cell lines (Methods) and supplies kinetic data. The Pearson correlation is used to compare the model-based sensitivity scores to the experimentally based sensitivity scores measured under the *in vitro* condition by Holbeck et al. [31]. Among these signaling agents, the dasatinib (0.964, p = 2.78e-03), everolimus (0.929, p = 6.75e-03), imatinib (0.893, p = 1.23e-02), and sunitinib (0.821, p = 3.41e-02) have high correlations of sensitivity scores (>0.80, Fig. 3a-c, Additional file 2). We also quantified the correlation between everolimus and temsirolimus treatment response of CRC cell lines measured by GI-50 and the model-based sensitivity score using the R^2^ measure. Under everolimus, R^2^ = 0.9713, and under temsirolimus, R^2^ = 0.9824. (Fig. 3d). These high correlations provide evidence that the NSAID-miR model captures drug effects in the CRC cellular system.

**Table 3:** Ten signaling agents and their targets with dissociation constant

Drug	Target	Dissociation constant (nM)
**Dasatinib**	ABL1	0.53
	EPHA3	0.09
	EPHA5/8	0.24
	PDGFRA	0.47
	LYN	0.57
	KIT	0.62
	SRC	0.21
**Erlotinib**	EGFR	0.67
	ERBB4	230
	LYN	530
	SRC	700
**Everolimus**	MTOR	2.2
**Gefitinib**	EGFR	1.0
	ERBB2	3500
	ERBB4	410
	LYN	990
**Imatinib**	ABL1	12.0
	ABL2	10.0
	KIT	14.0
	PDGFRA	31.0
	PDGFRB	14.0
**Lapatinib**	EGFR	2.4
	ERBB2	7.0
	ERBB4	54.0
**Nilotinib**	KIT	22
	PDGFRB	22
**Sorafenib**	DDR1	1.5
	DDR2	6.6
**Sunitinib**	FLT3	0.47
	KIT	0.37
	PDGFRA	0.79
	PDGFRB	0.08
**Temsirolimus**	MTOR	2.2
	VEGFR	0.75

The experimentally measured dissociation constants of these signaling agents were mainly taken from Karaman et al. [35]

**Fig. 4:**
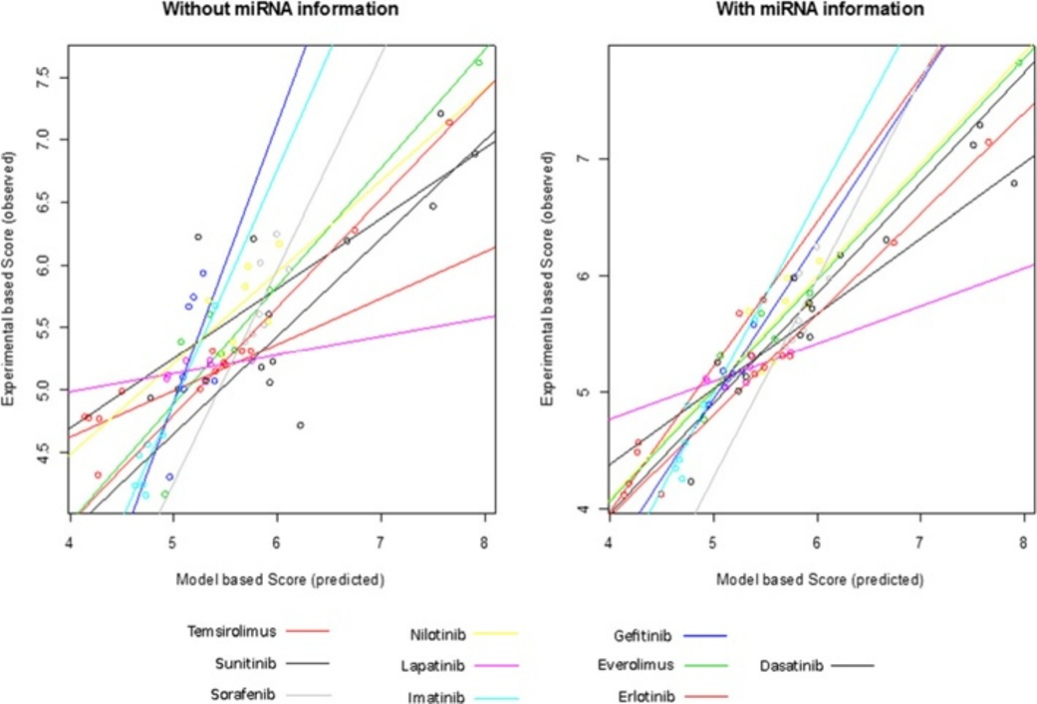
Single points represent reaction of a specific cell line under a specific treatment. Different colours represent different treatments**.** Overall correlation: without miRNA information 0.838, with miRNA information 0.947, p-value for difference in correlation structure given miRNA information (yes, no) p = 0.021

Lapatinib (0.679, p = 1.10e-02) has the lowest correlation of sensitivity scores (Fig. 3b, Additional file 2). The reason for this relatively low prediction rate could be that the inhibition effect of lapatinib in the NSAID-miR is only determined by the inhibition of three members of the ERBB-family. The dissociation constant between lapatinib and ERBB4 is relatively high (54 nM), which further weakens the effect of lapatinib in the NSAID-miR (Table 3). The overall correlation of the sensitivity scores of all ten drug treatments is 0.947. However, without use of miRNA expression data as input data for the *in silico* model, the same approach only achieved an overall correlation of 0.838 (Fig. 4). This difference (p-value: 0.021) indicates the value of miRNA expression profiles in better understanding the molecular mechanisms of cellular systems and their essential role in the prediction of therapeutic responses.

### Therapeutic Prediction of CRC Patients with Microsatellite Instability (MSI) and Microsatellite Stability (MSS)

Microsatellites are short repetitive DNA sequences that are prone to frameshift mutations and base-pair substitutions during duplication. MSI is one of the most extensively investigated genetic phenotypes, and is detected in approximately 15 % of CRC cases [36]. Many studies provide evidence that CRC with MSI status is associated with favorable clinical outcomes [37–41]. In contrast, other studies show controversial data related to the predictive value of MSI [42–45]; clearly, there is uncertainty as to the predictive ability of this genetic phenotype in clinical practice. In response to this, we apply the NSAID-miR model to predict the therapeutic responses of MSI/MSS patients. Moreover, we investigate the molecular mechanisms leading to the discrepancy between the clinical outcomes of individual colon cancer patients with MSI status versus those with MSS status. From the Cancer Genome Atlas [46], we obtained genetic data, including miRNA/gene-expression data and patient information (age, sex, race, cancer stage and other) of 22 colon cancer patients (Additional file 3), of which eleven have MSS status, and eleven MSI; the patients' cancer stages range from I to III. Further, two recent studies demonstrated that both drugs sirolimus and LY294002 (targeting mTor- and PI3K-signaling pathways, respectively) clearly reduced the growth of MSI tumors, but not MSS tumors [47, 48]. In order to investigate this issue with the application of the NSAID-miR model, we initialized the model with the gene-expression and miRNA-expression data of these patients individually (Methods). Subsequently, we performed the FCA to predict how these individual patients would respond to drug treatments.

**Fig. 5:**
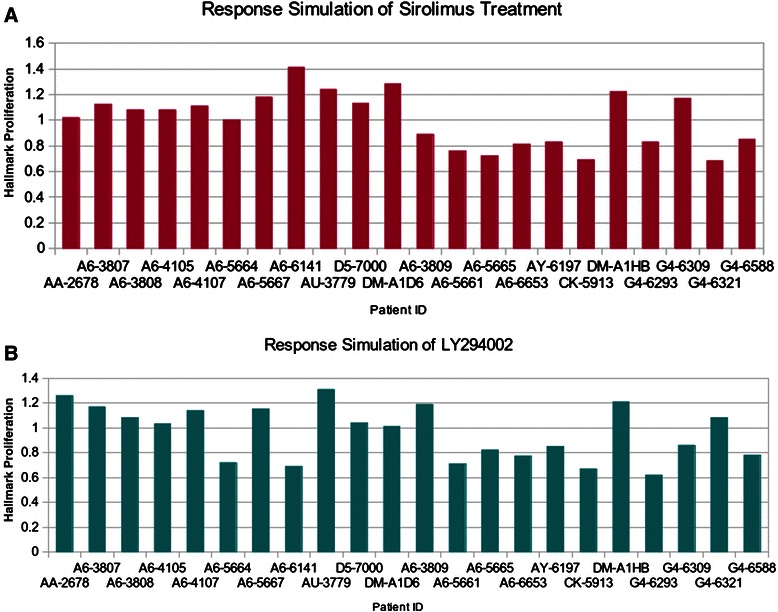
Simulated response of drug treatments. The model component 'proliferation' is considered as the readout component of this FCA analysis, which compares the flux from treatment state with the flux from control state of each patient. Patients with hallmark proliferation smaller than 1 are considered responders, while those with hallmark proliferation bigger than 1 are considered non-responders

**Fig. 6:**
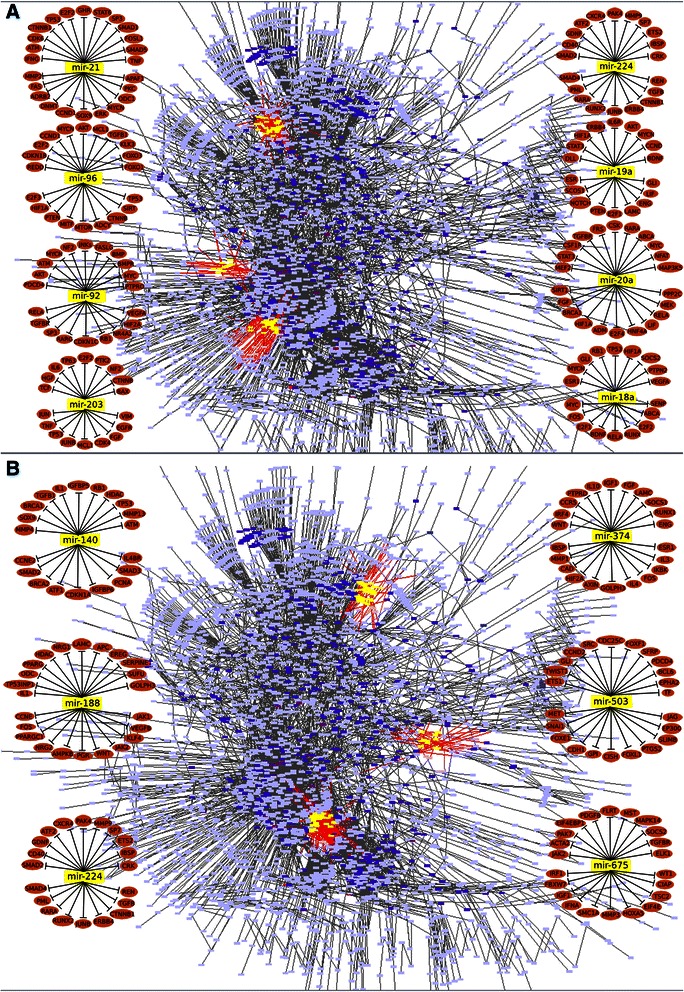
Simulated miRNA expression pattern after treatments. The simulated result reveals that global miRNA expression profile can be changed due to the drug treatment. According to the expression patterns, both treatments have more significant impact on the MSI patients than MSS patients. However, specific patients may have individual treatment responses

The results show that all MSS patients would not respond to the sirolimus treatment, regardless of colon cancer stage (Fig. 5). Among the eleven MSI patients, we found that two patients with advanced cancer stage III would not respond to the sirolimus treatment, while the remaining nine MSI patients would. In general, the predictions are in agreement with the results from the aforementioned studies [47, 48]. Further, our results support the finding that the prognosis for MSI tumors in stage III CRC is poor [49]. Taking model based miRNA level as readouts of model NSAID-miRNA, FCA results between MSI and MSS patients after treatment show that the expression levels of miR-18a, −19a, −203, −224, and −92 are downregulated on average by >15 % (p = 3.49e-06) in patients with stages I and II. The expression levels of miR-181b, −183, −20a, −21, −31 and −96 are downregulated on average by >15 % (p = 6.09e-04) in patients with stage III (Fig. 6a; Additional file 4). The expression levels of miR-30a, −143, −145, −200b and −378 are upregulated on average by >1.8 fold (p = 7.84e-06) in patients of all stages (Additional file 4).

**Fig. 7:**
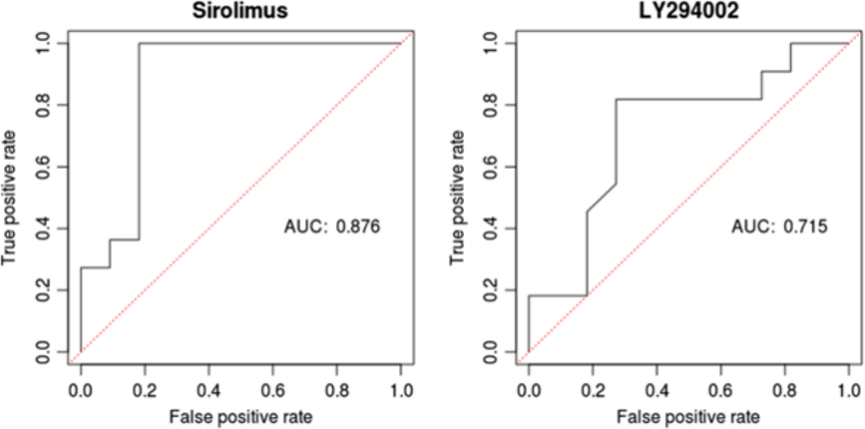
ROC analysis of discrimination of treatment responses between MSS and MSI patients

Two male African Americans in cancer stages IIA and IIIC among eleven MSS patients would respond to the LY294002 treatment. Further investigation of the FCA result shows that the expression levels of mir-21, −140, −188, −216, −224, −374, −503 and −675 were reduced in these two patients after treatment (Fig. 6b; Additional file 4). Eight among eleven MSI patients would respond to the LY294002 treatment. Interestingly, we found that the three MSI patients with CRC stage III, who would not respond to the LY294002 treatment, showed high activity of the cell-cycle pathway, and the expression levels of mir-21, −34a, −95, −135a and −320 remained nearly unchanged after this treatment (Additional file 4). This result might reveal the key miRNA-regulators that negatively contribute to the clinical outcome of MSI patients. Furthermore, by using a ROC curve and the corresponding AUC, we quantified the discrimination between response between MSS and MSI patients through the *in silico* prediction given by our model. The AUC for response prediction under sirolimus is 0.876, The AUC for response prediction under LY294002 is 0.715. (Fig. 7)

## Conclusion & Discussion

This study introduces a concept which integrates different types of molecular data for individualized medicine. It uses an *in silico* environment to capture the molecular regulation effect of miRNAs within individual cancerous cellular systems (Fig. 2). Four major sources of input information are used to calculate the individual response of the system: molecular, kinetic, individual genetic (miRNA/mRNA expression data) and treatment data. The internal network structure of the NSAID-miR model covers molecular signaling pathways (including transcription and translation, protein-protein interaction, and protein modification) and miRNA regulation. The kinetic data describes kinetic values of different types of reactions (such as transcription, phosphorylation, complex formation, receptor-ligand-binding) and allows to implement the treatment effects. The kinetic data impacts the signal flow (defining classical chemical reactions of substrates for producing products with or without modifiers) throughout the network. Contrasting the flux in the untreated cell with the flux of the treated cell allows quantifying changes in the cancer hallmarks. These changes can be used to predict treatment response of the system.

During this study, data of CRC cell lines as well as patients were used for the validation. For ten agents, we simulated the responses of seven CRC cell lines and compared them to their *in vitro* drug response data. There is high correlation, which indicates the reliability and precision of the predictions of the proposed model. In order to give a first demonstration of the potential clinical usefulness of this concept, we received the miRNA and gene expression data of 22 MSI/MSS patients provided by the Cancer Genome Atlas [46] for predicting the clinical outcome of the sirolimus and LY294002 treatments. The prediction results show that most MSI patients would respond to both drug treatments, however most MSS patients would not. At the moment, data on clinical response for these patients is still not available. But our result is in accordance with clinical knowledge that MSI status is related to the response of the treatment under study [47,48]. Based on our results, we strongly hypothesize that one molecular reason for better therapeutic outcomes of MSI patients could be the upregulation of tumor-suppressor miRs and downregulation of oncogenic miRs, which drives the cellular system of patients with MSI status away from the full-fledged malignant cellular state with strong drug resistance and uncontrolled proliferation.

In a recent study, Ellwanger and colleagues [50] decipher the role of miRNA on a large scale, which provides knowledge for the implementation of miRNA regulation in molecular *in silico* models. We see that many of their findings are already implemented in our NSAID-miR model, for instance, the regulation mechanisms of mir-21, mir-181 and let-7. However, the NSAID-miR model might be the first molecular signaling model which contains not only validated miRNA-target relationship information but also includes literature-referenced relationships between transcription factors and miRNAs. In addition, the NSAID-miR model can be applied to investigate therapeutic response of patients with cancers beyond CRC. For instance, we are studying a genome-scale model of acute myeloid leukemia (AML) to predict individual response to AML clinical treatments; the results achieved thus far are promising (data not shown), which indicates that our approach also possesses the potential to be extended to diverse other cancer types. However, one limitation of our model is the applied kinetic data, which is mainly determined through empirical experience. How to perform appropriate estimates in patient groups (depending on age, sex, etc.) is the issue of our future research. Furthermore, our concept does not consider metabolic molecular information. This is another challenge of future research.

## Additional file

Additional file 1:
**Validated miRNA target with literature reference.**

Additional file 2:
**Experimentally based sensitivity score and model-based sensitivity score for treatments of ten signaling agents with and without consideration of miRNA expression data.**

Additional file 3:
**Patient Information.**

Additional file 4:
**Comparison result between treatment state and control state from the FCA analysis.**
